# Effects of concentrate supplement on camel performance, forage utilization, and energy usage in arid-area rangelands

**DOI:** 10.1186/s12917-025-04833-6

**Published:** 2025-05-28

**Authors:** Ahmed R. Askar, Ahmed R. Saber, Sabbah Allam, Khaled Z. Kewan, Mohammed H. Bakr, Faysal Fayed, Hamedi M. Kandil, Samy Abo Ragab, Basma A. Hussien, Samir S. Abou El Ezz, Mohsen M. Shoukry

**Affiliations:** 1 Animal and Poultry Nutrition Department, Desert Research Center, Cairo, Egypt; 2 Animal Production Department, Faculty of Agriculture, Cairo University, Giza, Egypt; 3 Economic Department, Desert Research Center, Cairo, Egypt; 4 Animal and Poultry Physiology Department, Desert Research Center, Cairo, Egypt; 5 Animal Production Department, National Research Centre, Cairo, Egypt

**Keywords:** Camels, Arid-area rangeland, Supplementation, Forage utilization, Distance traveled, Energy expenditure

## Abstract

**Background:**

In light of climate change, camels, as drought-resistant animal species, have become a crucial component of the pastoralists' economy, ecology, and culture. They exhibit an extraordinary capacity to adapt to harsh environments. Most camels rely on grazing on natural rangelands to meet their daily nutritional requirements. Climate and watering intervals influence the foraging behavior of camels, as well as forage quality and availability. This experiment investigated the effects of adding a concentrate supplement (CS) on camel performance, forage consumption and digestibility, and energy usage in arid-area rangelands during the dry season.

**Methods:**

Eighteen dry and non-lactating she-dromedary camels, aged 8–10 years and weighing an average of 438 ± 5.7 kg, were employed in the experiment. Camels were randomly selected from a herd of 120 animals, individually marked for identification, and divided into three groups of six. The CS was administered separately before grazing at 0% (control), 50% (low), and 100% (high) of the metabolizable energy requirements for maintenance. The herd grazed daily from 08:00 to 18:00 in an area dominated by *Ababasis articulate*, a low-quality forage containing more than 70% neutral detergent fiber.

**Results:**

Adding a high CS level alleviated animal deterioration by reducing weight loss from − 1049 to − 192 g/day and significantly increasing dry and organic matter digestibility. A substantial drop in forage consumption, accompanied by a significant adverse effect on fiber digestibility (*P* < 0.01), was observed with CS addition. The Global Positioning System results revealed that the high CS addition considerably reduced (*P* < 0.01) the distance traveled from 25 to 13 km, leading to lower (*P* < 0.01) walking and grazing activity and higher (*P* < 0.01) standing and resting time. The results were consistent with energy expenditure data, reflected in a more incredible retained energy for high vs. low or control levels of the concentrate.

**Conclusions:**

It is recommended that camels be confined and not allowed to graze, or they graze for a shorter period of time during drought seasons when palatable forage is scarce. Supplementary feeding is essential to maintain camels in arid-area rangelands. When CS is utilized, the interdependent effects on forage utilization must be considered. The CS should be used under restriction or replaced partially or completely with high-quality forage during the drought season.

## Introduction

In light of climate change, camels, as drought-resistant animal species, have become an important aspect of pastoralists'economy, ecology, and culture [1]. They have an extraordinary capacity to adapt to harsh environments [2]. Most camels rely on grazing on natural rangelands to meet their daily nutritional requirements, while their foraging behavior is influenced by climate, watering intervals, and forage type and availability [3, 4]. In arid environments and poor conditions, camels may travel 50–70 km daily in search of feed [3], expending a large amount of energy [5] and increasing their energy needs [6, 7]. Given that energy is more critical for camels than protein requirements [8], the energy cost of grazing activity (ECA) has been reported to consider a significant proportion of the total energy expenditure [9, 10]. Beker et al. [5] discovered that the ECA increased by 5.79% and 5.05% of the metabolizable energy used for maintenance for each hour spent grazing/eating or grazing/eating and walking, respectively.

In this context, proper feeding practices are closely related to rangeland management, including establishing policies for the strategic use of supplementation during critical stages when the animal’s nutrient requirements exceed the supply from forage. Supplementation may be required for animal survival throughout the dry seasons, even though it may raise the feeding cost [11]. It may partially replace grazed forage consumption, affecting diet composition [12] and digestibility [10, 13]. It can also reduce grazing time [14, 15], decrease ECA [5], and improve feed consumption efficiency [10, 15]. However, discrepancies were found in the responses of animal species to the level of supplementation when grazing arid-area rangelands. In goats, increasing the concentrate supplement from 1 to 2% of body weight negatively impacted forage intake and digestibility. In contrast, in sheep, it enhanced forage intake and digestibility, including the digestion of dry matter, organic matter, and fiber fractions [10]. A better understanding of feeding behavior allows for the development of management strategies that maximize the utilization of ecosystems for enhanced livestock production [16]. We hypothesis that camels require supplementation to maintain or perhaps survive when grazing arid area rangeland during the drought season.

This experiment aimed to study the effects of adding concentrate supplementation on camel performance, forage consumption, and energy usage in arid-area rangelands. The expected findings help make appropriate decisions, facilitate management practices, enhance animal productivity, and avoid the deterioration of the fragile, dry pastoral system.

## Materials and methods

### Study area

The experiment was conducted in the “National Campaign for the Promotion of Camel Productivity” study area, Desert Research Center, Egypt, in collaboration with the Bedouins. The study area is on the North West Coast of Egypt, Sidi Barrani, approximately 40 km from Marsa Matruh City, Matrouh Governorate, Egypt, at latitude 31° 21′ 952″ N and longitude 27° 00′ 490″ E. It is an arid desert area on the Mediterranean Coast of Egypt. There is virtually no rainfall throughout the year. The average outside temperature was 23.8 °C, the average humidity was 70.5%, and the annual rainfall was 148 mm [17].

### Animals and treatments

Eighteen dry and non-lactating she-dromedary camels, aged 8–10 years and weighing an average body weight (BW) of 438 ± 5.7 kg, were used to investigate the effects of concentrate supplement (CS) addition on camel performance and forage utilization in arid-area rangelands throughout the drought season. The camels were selected from a herd of 120 animals and individually marked for identification before being herded with the rest of the herd. They were distributed into three treatments, each with six, and the CS was separately given in separated feeders before grazing at 0% (control), 50% (low), and 100% (high) of the metabolizable energy (ME) used for maintenance (MEm, 467.0 kJ/kg BW^0.75^, [18]). They individually received the CS as per treatment before the commencement of grazing. The herd was grazing the arid-area rangelands daily from 08:00 to 18:00, dominated by *Ababasis articulate*. The proximate analysis of the CS and *Ababasis articulate* is shown in Table 1.

**Table 1:** The chemical composition of concentrate supplement and *Ababasis articulate*, based on a dry matter (DM) basis

Ingredients	Concentrate Supplement^a^	*Ababasis* *articulate*	
**Dry matter,** g/kg fresh matter	921		591
**Gross energy,** MJ/kg DM	16.7		12.5
**Chemical composition,** g/kg DM			
Organic matter	903		797
Crude protein	134		64
Neutral detergent fiber	325		743
Acid detergent fiber	119		379

^a^The supplement contained 55% corn, 15% soybean meal, 10% cottonseed meal, 15% wheat bran, 2.5% limestone, 1.5% salt, 0.5% sodium bicarbonate, 0.1% yeast, 0.1% antitoxins, and 0.3% minerals and vitamins premix

### Experimental procedures

The study lasted one and a half months during the dry season, including two weeks for measurement. The entire herd grazed in the arid-area rangelands from 08:00 to 18:00; they then went back to stay in the barn at night.. The camels were weighed monthly. However, no water was available in the grazing area, so camels had to walk about 13 km to a watering point (constructed cistern) twice a week to access free water, which was not measured.

In the third and fourth weeks of the experimental period, some approaches [7] had been employed to monitor grazing behavior. Visual observations were used to determine the time spent grazing or eating, walking without grazing, standing, and lying. Walking time is at a relatively fast pace, presumably with no grazing, whereas lying time is solely without grazing. In this regard, three people monitored three different camels every day, one per each treatment, for six consecutive days in the third week, with a total of six animals per treatment by the end of the week. In the fourth week, five camels within each group were provided with the RCX3 monitors (Polar Electro Oy, Finland) connected with the Global Positioning System (GPS) that was employed to make a tracking map of the flock and determine the animal's distribution and grazing, and drinking paths in the rangeland area. A unique ID number for each camel was assigned to allow for precise tracking maps over satellite image. The daily distance traveled was also individually calculated.

Furthermore, the 3D satellite imagery was used to analyze the grazing area's topography. A Digital Elevation Model (STRM DEM) with a spatial resolution of 30 m was downloaded from the United States Geological Survey's (USGS) platform [http://earthexplorer.usgs.gov/]. The topography features, including elevation above sea level, slope direction, and gradient, were analyzed, using ArcGIS software. In addition, the natural vegetation cover of the grazing area was estimated using Sentinel-2 satellite imagery with a 10-m resolution for land use/land cover analysis. The data can be available through the [Sentinel-2 10-Meter Land Use/Land Cover] link [19].

During the measurement period, which lasted the fifth and sixth weeks of the experiment, five animals per treatment were provided with fecal bags, and three days were allowed to adjust to the new condition before feces collection began for the next seven days. During the seven-day collecting period, the bags were emptied twice daily: in the morning (06:00) and afternoon (18:00). The whole fecal output was collected every day, and a 10% sub-sample of each animal was taken and aggregated into individual composite samples across the seven-day collection period. Simulated grazed samples were also obtained every day. The feces and forage samples were air-dried at 55 ºC for 48 h and stored for subsequent analysis. The acid-insoluble ash was used to estimate the forage intake and digestibility [11] based on the following equations:

1. The amount of internal marker in feces = The amount of internal marker in intake 
2. Fecal output * %internal marker in feces = Total intake * %internal marker in intake 
3. Total intake = (Fecal output * %internal marker in feces) / %internal marker in intake

However, the digestibility (%) was calculated as follows:

4) Digestibility (%) = (Total intake – Fecal output) * 100/ Total intake 

Please note that the total intake equals the sum of the known CS and estimated forage intakes.

### Energy expenditure

RCX3 heart rate (HR) monitors (Polar Electro Oy, Finland) were used to estimate energy expenditure (EE) over 48 h. Heart rate data were collected at one-minute intervals. The EE:HR ratio was derived from a calibrated camel study [4] using an open-circuit respiratory system [20]. The Polar software was used to analyze the data. The EE was calculated daily from the EE: HR ratio.

The gross energy (GE) was measured using a bomb calorimeter (IKA, model C 200, Germany), with benzoic acid as the standard. The ME was calculated as 82% of digestible energy (DE, [6]). The difference between ME intake and total EE estimates the energy balance (EB).

### Weather data

Outside ambient temperature (T °C) and relative humidity (RH) were measured daily with 20-min intervals (RC-4HA Temperature and Humidity Data Logger). The temperature–humidity index (THI) was estimated as follows: (0.8 × T) + [(RH/100) x (T—14.4)] + 46.4 [21].

### Analytical procedures

Feeds, orts, and feces samples were analyzed for dry matter (DM), organic matter (OM), GE, crude protein (CP, [22]), neutral detergent fiber (NDF, [23]) and acid detergent fiber (ADF, [22]), using the filter bag, ANKOM Technology Corp., USA. The AIA was analyzed according to the method of Van Keulen and Young [24].

### Statistical analysis

Data were analyzed using the GLM procedure of the SAS statistical package [25], with a one-way analysis model consisting of the impact of supplementation. The model was *Yij* = *μ* + *Si* + *eij*, where *Yij* is the dependent variable, *μ* the overall mean, *Si* is the fixed level of CS effect, and *eij* is the residual. Differences between means are significant when the *P*-value is below 0.05 but considered a tendency when the *P*-value is between 0.05 and 0.10.

## Results

**Fig. 1:**
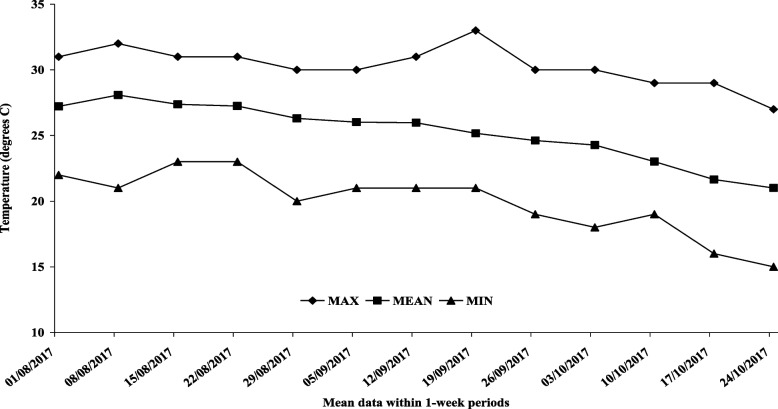
Mean, maximum, and minimum temperature were set in 1-week periods throughout the experimental period, during which camels were exposed to August- October

**Fig. 2:**
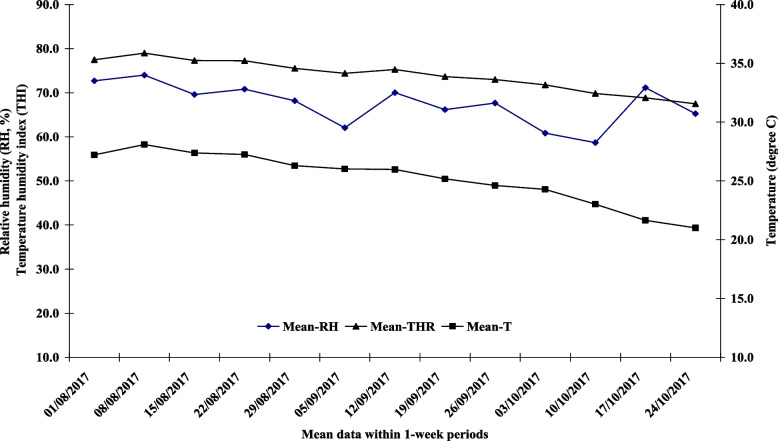
Mean temperature, relative humidity (RH), and temperature–humidity index (THI) were set in 1-week periods, during which camels were exposed to August- October

Mean T, RH and THI were obtained daily. Average mean, low and high values for T were 25.2 ± 0.62 °C, 19.9 ± 0.68 °C and 30.3 ± 0.41 °C, respectively (Fig. 1), while mean RH and THI were 67.5 ± 1.30% and 73.9 ± 1.00, respectively (Fig. 2).

### Animal performance

The effects of adding CS on BW changes are shown in Table 2. Although similar initial BW was observed across groups, the BW changes was significantly higher (*P* < 0.01) among treated groups. Adding supplementary feeding alleviated the animals'deterioration by reducing weight loss from − 1049 g/day to − 558 g/day or − 192 g/day when CS was administered at 50% or 100% of the MEm, respectively.

**Table 2:** Effects of different concentrate supplement levels on body weight changes and feed intake by non-productive she-camels grazing in the arid-area rangelands

Item	Concentrate supplement level			SEM	Significant
	**Control**	**Low**	**High**		
**Body weight,** kg					
Initial	437.8	438.8	437.4	10.6	ns
Final	406.3	422.1	431.6	10.9	ns
**Body weight change,** kg	−31.5^c^	−16.7^b^	−5.8^a^	0.91	***
**Average daily gain,** g/d	−1049^c^	−558^b^	−192^a^	30.5	***
**Forage intake**					
g dry matter/day	3523^a^	2672^b^	1578^c^	178.6	***
g dry matter/kg BW^0.75^	38.9^a^	28.7^b^	16.7^c^	1.581	***
**Total intake**					
g dry matter/day	3523^b^	3972^a^	4230^a^	205.2	*
g dry matter/kg BW^0.75^	38.9^b^	42.6^a^	44.7^a^	1.629	*

^a,b,c^Means having different superscripts within the same row differed significantly(*P* < 0.05), otherwise no significant differences were detected, *ns* Non-significant; * = *P* < 0.05, ***P < 0.001; *SEM* Standard error of means; Concentrate supplement levels: Zero% (control), 50% (Low) and 100% (High) of the metabolizable energy requirements used for maintenance (MEm)

The effects of CS levels on forage consumption and nutrient digestibility are presented in Tables 2 and 3. The addition of CS negatively impacted (*P* < 0.01) forage consumption; however, it had a significant beneficial effect (*P* < 0.01) on overall DM intake and digestibility (Table 3), consistent with the observed BW changes (Table 2). Nevertheless, NDF and ADF digestibility was significantly reduced (*P* < 0.01) by the addition of CS, coinciding with the lower forage intake (Table 2).

**Table 3:** Effects of different concentrate supplement levels on intake and digestibility by non-productive she-camels grazing in the arid-area rangelands

Item	Concentrate supplement level			SEM	Significant
	**Control**	**Low**	**High**		
**Body weight (BW)**					
kg	406	422	432	10.9	ns
**Dry matter**					
Intake, g/day	3523^b^	3972^a^	4230^a^	205.2	*
Intake, g/kg BW^0.75^	38.9^b^	42.6^a^	44.7^a^	1.629	*
Digestion, %	44.6^c^	52.4^b^	57.8^a^	1.59	***
**Organic matter**					
Intake, g/day	2809^b^	3233^a^	3509^a^	165.3	**
Intake, g/kg BW^0.75^	31.0^b^	34.7^a^	37.1^a^	1.31	**
Digestion, %	44.4^c^	55.6^b^	61.2^a^	1.64	***
**Crude protein**					
Intake, g/day	224.6^c^	334.6^b^	435.6^a^	15.39	***
Intake, g/kg BW^0.75^	2.50^c^	3.58^b^	4.60^a^	0.119	***
Digestion, %	39.4^c^	56.4^b^	65.6^a^	2.95	***
**Neutral detergent fiber**					
Intake, g/day	2615^a^	2381^a^	1982^b^	139.4	**
Intake, g/kg BW	28.9^a^	25.5^b^	20.9^c^	1.18	***
Digestion, g/kg	58.2^a^	53.2^b^	51.0^b^	1.22	***
**Acid detergent fiber**					
Intake, g/day	1336^a^	1160^b^	896^c^	70.3	***
Intake, g/kg BW	14.7^a^	12.4^b^	9.5^c^	0.60	***
Digestion, g/kg	48.0^a^	42.7^b^	40.8^b^	1.47	**

^a,b,c^Means having different superscripts within the same row differed significantly(*P* < 0.05), otherwise no significant differences were detected, *ns* Non-significant; * = *P* < 0.05, **P < 0.01; ***P < 0.001; *SEM* Standard error of means; Concentrate supplement levels: Zero% (control), 50% (Low) and 100% (High) of the metabolizable energy requirements used for maintenance (MEm)

### Grazing activity

**Fig. 3:**
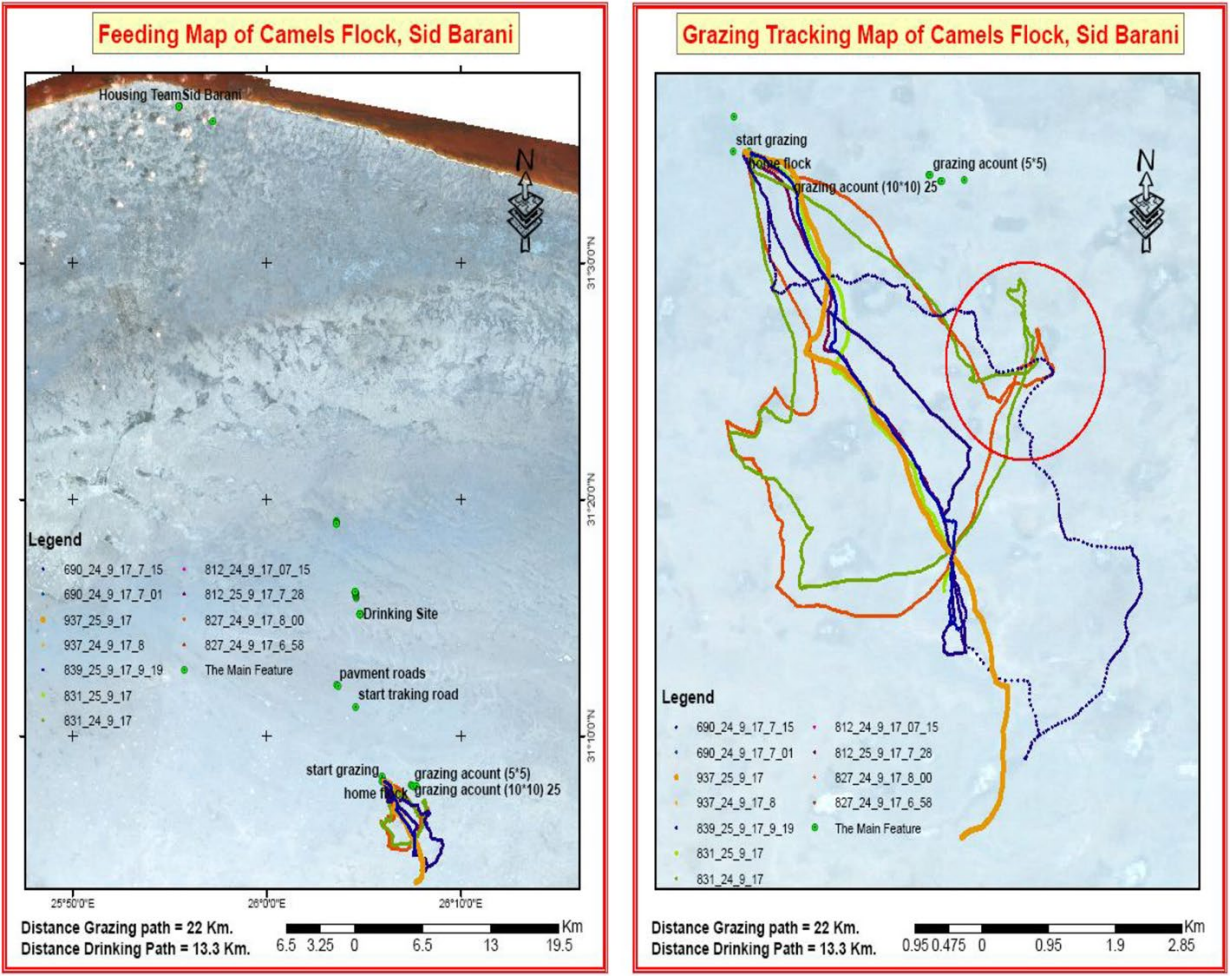
Grazing and drinking path during the drought season, looking for feed and water, revealed from the Global Positioning System (GPS) data over the satellite image

The effects of adding CS on grazing activity are shown in Fig. 3 and Table 4. The GPS results revealed that camels in the control group (zero supplementation) walked approximately 25 km daily in search of feed and nearly 27 km every three days to access water (drinking path = 13.3 km, the distance between camel pens and the water source). CS addition considerably reduced this distance, from 25 to 21 km or 13 km for low and high supplementation levels, respectively (Fig. 3 and Table 4). This was associated with significantly reduced (*P* < 0.01) walking and grazing activity and increased (*P* < 0.01) standing and resting time (Table 4).

**Table 4:** Effects of different concentrate supplement levels on grazing behavior by non-productive she-camels grazing in the arid-area rangelands

Item	Concentrate supplement level			SEM	Significant
	**Control**	**Low**	**High**		
**Distance travel,** km/day	25.2^a^	21.0^b^	13.2^c^	0.77	***
**Grazing activity**					
**Grazing hours/day**	8.1	8.0	8.0	0.18	ns
**Minutes/day**					
Walking	39.5^a^	23.7^b^	7.8^c^	2.54	***
Grazing	428.5^a^	351.2^b^	349.2^b^	14.84	**
Standing	17.2^c^	66.5^a^	40.7^b^	4.29	***
Lying/resting	0.8^c^	37.8^b^	84.0^a^	3.35	***
**% of total time/day**					
Walking	8.2^a^	4.9^b^	1.6^c^	0.55	***
Grazing	88.1^a^	73.1^b^	72.6^b^	1.68	***
Standing	3.5^c^	14.0^a^	8.5^b^	0.98	***
Lying/resting	0.2^c^	8.0^b^	17.3^a^	0.89	***

^a,b,c^Means having different superscripts within the same row differed significantly(*P* < 0.05), otherwise no significant differences were detected, *ns* Non-significant; ** = *P* < 0.01, ***P < 0.001; *SEM* Standard error of means; Concentrate supplement levels: Zero% (control), 50% (Low) and 100% (High) of the metabolizable energy requirements used for maintenance (MEm)

### Topography features and asseing vegetation cover

**Fig. 4:**
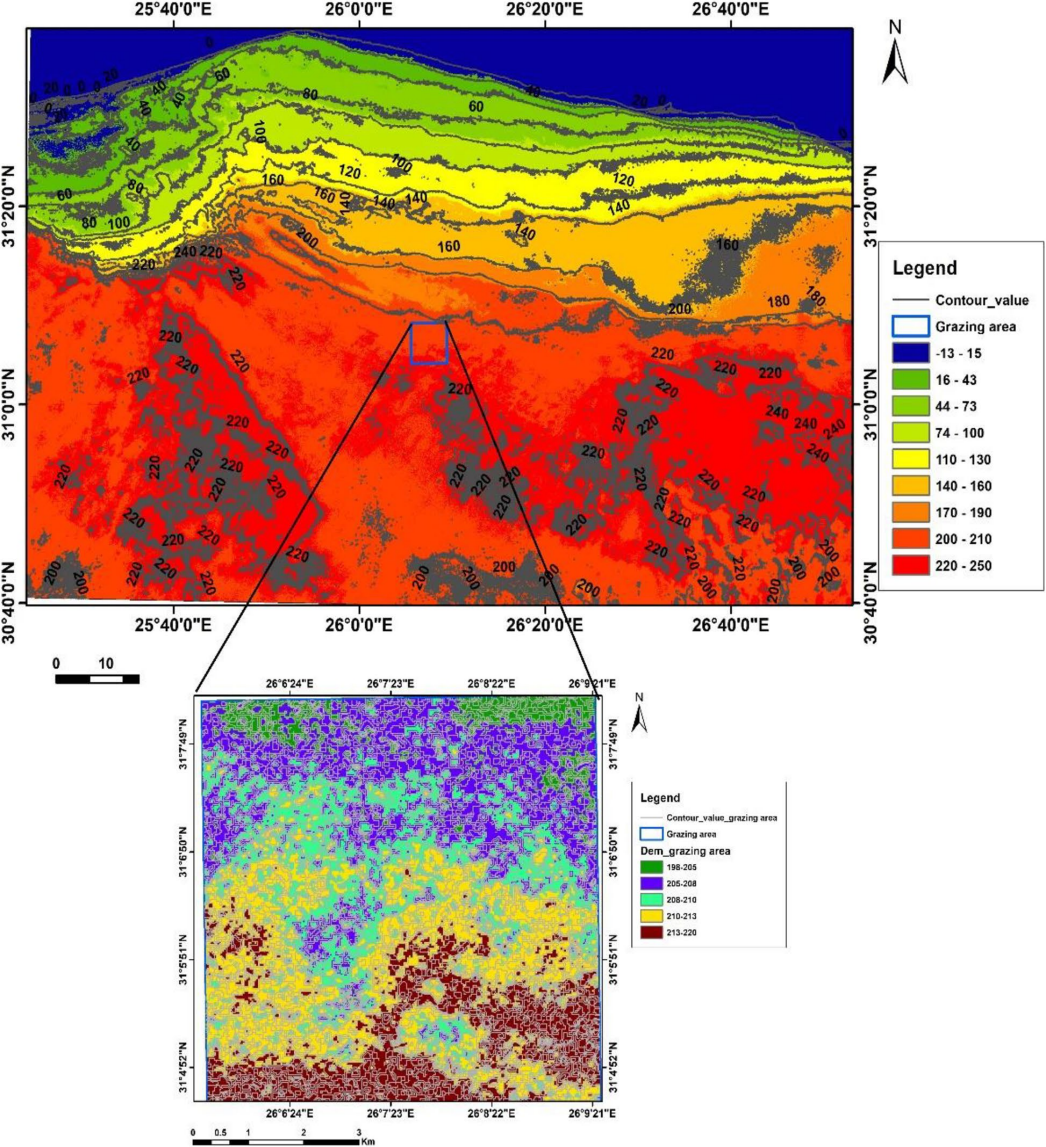
The surface topography of the grazing area using a Digital Elevation Model (DEM) generated from the United States Geological Survey (USGS) platform, using ArcGIS software

The elevation in the region ranges from −13 to 250 m above sea level (Fig. 4), while the specific grazing area is from 198 to 220 m. The region exhibits a steep slope from the north to the sea, with shallow soils that are rocky and stony. The natural vegetation cover accounts for around 10% of the overall ground surface, which, together with the topography features, reflects the physical effort required by camels to access food or water resources in the grazing area.

### Energy utilization

The results of energy utilization are shown in Table 5. The gross and digestible energy intakes (MJ/d) followed the same trend as OM intake and digestibility. However, non-supplemented camels exhibited significantly higher (*P* < 0.01) total EE compared to supplemented camels (Table 5). In this context, differences in estimated EE among camels receiving varying CS levels were evidente throughout the grazing period, as illustrated by the GPS results in Fig. 3 and Table 4. The EB was negative and significantly greater (*P* < 0.01) for camels receiving the high CS level, followed by those receiving the low, with the lowest being the zero level. This was attributed to increased ME intake (*P* < 0.01) and reduced EE as the CS level increased.

**Table 5:** Effects of different concentrate supplement levels on energy utilization by non-productive she-camels grazing in the arid area rangelands

Item	Concentrate supplement level			SEM	Significant
	**Control**	**Low**	**High**		
**Energy utilization**^**d**^**:**					
GE (MJ/d)	44.0^c^	53.7^b^	61.3^a^	2.68	***
GE (kJ/kg^0.75^)	486^c^	576^b^	647^a^	20.6	***
DE (MJ/d)	15.4^c^	27.9^b^	37.5^a^	1.50	***
DE (kJ/kg^0.75^)	170^c^	299^b^	397^a^	13.4	***
DE (%)	34.9^c^	51.9^b^	61.2^a^	1.37	***
ME (MJ/d)	12.6^c^	22.8^b^	30.8^a^	1.23	***
ME (kJ/kg^0.75^)	139^c^	245^b^	325^a^	11.0	***
HR (Beat/min)	60.0^a^	55.3^b^	52.2^c^	0.95	***
EE (kJ/kg^0.75^)	432^a^	398^b^	376^c^	6.67	***
EE/ME ratio	2.92	1.51	1.06		
EB (kJ/kg^0.75^)	−293^c^	−153^b^	−50.6^a^	10.95	***

^a,b,c^Means having different superscripts within the same row differed significantly(*P* < 0.05), otherwise no significant differences were detected, ***P < 0.001; *SEM* Standard error of means; Concentrate supplement levels: Zero% (control), 50% (low) and 100% (high) of the metabolizable energy requirements used for maintenance (MEm)

^d^*GE* Gross energy, *DE* Digestible energy, *ME* Metabolizable energy, *HR* Heart rate, *EE* Energy expenditure, *EB* Energy balance

## Discussion

### Camel performance and forage utilization

Under desert conditions with a restricted water intake, the BW changes reflected observed changes in the intake and nutrient digestibility. Adding a high CS level alleviated camel deterioration by reducing their daily weight loss from − 1049 g to − 192 g per day (Table 2), supported by a significant increase in total DM intake (Table 2) and digestibility (Table 3). This suggests that supplemented camels were provided with a ration low in fiber and high in readily available carbohydrates, which are more efficiently utilized for rumen microbial digestion [26, 27]. This demonstrated that supplementary feeding is essential to maintain grazing camels throughout the drought seasons. The results align with previous studies on camels [4] and small ruminants [10, 11].

It is worth noting that the CS addition negatively impacted (*P* < 0.01) the roughage consumption that was substituted by concentrate and resulted in a greater total DM intake for supplemented vs. non-supplemented camels (Table 2). The findings are consistent with those observed in camels [28–30], sheep [4, 31] and goats [10, 12, 14]. Although non-supplemented camels ingested more roughage (Table 2), they could not cover their nutrient requirements [4, 32] as roughage consumption was restricted by its high NDF content (more than 70%, Table 1). The total DM intake ranges from 38.9 to 44.7 g/kg BW^0.75^ (Table 2), which is considered lower than the values reported by Gihad et al. [33] and Gauthier-Pilters [34]. Furthermore, Askar et al. [4] reported that the DM intake of camels was approximately 52.4–66.6 g/kg BW^0.75^ when they grazed a limited cultivated pasture and were supplemented with a concentrate.

Alkali et al. [35] and Askar et al. [4] concluded that plants'chemical and physical properties are the primary factors for forage selectivity by grazing camels. A higher NDF intake was reported to limit the ability to consume enough forage, which was linked to low intake capacity [36]. Roughage intake is always controlled by the rumen fill and rate of disappearance, both of which are influenced by the digestion and passage rate [37, 38]. However, accumulating indigestible portions of NDF leads to longer retention times, which are unavailable for microbial digestion, resulting in rumen fullness and restricted forage intake [39].

A significant drop in a roughage-to-concentrate ratio, 67.2 or 37.3%, for camels that received a low or high level of CS, rspectively (Table 2) was accompanied by a significant decline in fiber digestibility (Table 3). The results are in line with those published by Bhattacharya et al. [40], Askar et al. [4, 30] for camels, Askar et al. [10] for small ruminants, and Reis and Combs [41] for dairy cattle. The addition of CS was expected to have a negative impact on roughage consumption and fiber digestibility [13], probably attributed to rumen fermentation alterations [42]. This may be linked to its negative effect on rumen pH [26], which reduced the population and activity of cellulolytic bacteria [26, 43] and protozoa [44, 45], and might reduce the efficiency of microbial protein synthesis and consequently forage utilization [26, 27]. A significant reduction in roughage intake and fiber digestibility was found in camel calves receiving a CS at a high level of 1.3% of BW [30], which was associated with a considerable drop in rumen pH, similar to what Allam et al. [46] and Askar et al. [10] reported in goats grazing in arid-area rangelands and supplemented with different levels of CS.

The impact of CS on forage consumption varies depending on the quality and type of forage [11, 28, 47, 48], the CS level [10, 30] and the animal species [10]. No effect was found for supplementary feeding on forage consumption when the forage was of high quality [49, 50], while Askar et al. [11] found that CS is important for improving forage consumption and nutrient digestibility in sheep fed low-quality forage. However, Askar et al. [10] concluded that the effect of CS on roughage consumption varied between sheep and goats at a high CS level (2% of BW), while both were in similar status at a low CS level (1% of BW).

Farid et al. [28] found that camel calves performed better on *Atriplex* as a basal diet than clover hay (high-quality forage) or rice straw (low-quality forage); however, Askar et al. [51] found that sheep and goats performed poorly when they fed the same *Atriplex* with a negative energy and/or nitrogen balance. On the one hand, this may be related to the more salt required for camels, which is in higher proportion in *Atriplex*. Camels require seven times more salt than other animal species [52], and they, without regular access to salty feed, require about 40 g of salt per day [53]. On the other hand, in comparison to bovines, camel saliva contains a varying content of high molecular weight mucin-glycoprotein that confers protection to the mucosa of the digestive tract from mechanical injuries and fixes plant tannins by avoiding their detrimental effects on ruminal protein metabolism [54]. Moreover, *Atriplex* is considered a lush green plant, more palatable and preferred for camels than sheep and goats.

### Grazing behavior and energy utilization

The GPS results (Table 4 and Fig. 3) revealed that grazing camels without supplementation walked approximately 25 km daily in the arid-area rangelands. In comparison, adding a high level of CS considerably reduced this enormous distance traveled to 13 km, which was associated with notably less grazing and walking activity and longer resting while standing or lying (Table 4). Camels on natural rangelands rely on grazing to meet their daily nutrient requirements, whereas climate and watering intervals influence foraging behavior and activity and forage quality and availability [3, 4].

Camels could walk longer distances and spend more time grazing and foraging for feed [3]. This extra muscular activity may increase the camel's energy requirements [6, 7], causing them to expend a substantial amount of ECA [5, 10]. This suggests that grazing camels without supplementation throughout the dry season leads to expending more ECA [5] and, finally, severe animal deterioration [11]. The tough terrain characteristics, which include varying elevations above sea level (from 198 to 220 m) with rocky and stony surfaces (Fig. 4), as well as a low vegetation cover (nearly 10% of the overall ground surface) throughout the dry seasons, reflect the physical effort required by grazing camels to reach feed and water supplies in the study area.

The results align with the current findings of energy usage data, which indicated that adding CS increased ME intake while considerably reducing ECA and enhancing retained energy (Table 5) and animal performance (Table 2). Askar et al. [14] reported that the effects of CS on performance could include less energy used for maintenance due to shorter grazing time. Moreover, camels receiving a high CS level had a lower and more efficient EE/ME ratio, indicating better performance than non-supplemented camels (Table 5).

Camels in arid environments appear more susceptible to energy than protein deficiency [8]. The current study estimated the ECA to be 61% of the stated MEm [4] for non-supplemented camels. This value was reduced to 40% of the MEm for the high-supplemented camels group. The ECA was estimated to be a large portion of the total EE [5, 9]. Grazing activity was reported to account for a significant portion of the EE, nearly 65–73% of the reported MEm [10] when sheep and goats grazed in arid-area rangelands and were supplemented with different CS at 1 or 2% of BW. Beker et al. [5] discovered that the ECA increased by 5.79 and 5.05% of the MEm for each hour spent grazing/eating or grazing/eating with walking, respectively.

In this context, the addition of CS is required to maintain camel grazing in arid-area rangelands throughout the drought seasons [10, 11]. Although CS addition may increase feeding costs [11] and reduce forage consumption [12], it may alter diet composition [11], improve digestibility [10, 13], minimize grazing time [14, 15] and ECA [5], and improve feed consumption efficiency [15].

On the other hand, forage quality has been reported to be another factor affecting total EE [6]. *Ababasis articulate* is a poor-quality forage, with more than 70% NDF and less than 6.5% CP (Table 1). This poor-quality forage was expected to have a negative impact on foraging behavior [4] and animal survival throughout drought seasons [11, 38, 55].

Small ruminants cannot sustain an energy and/or nitrogen balance when fed low-quality forage, whether indoors [20, 56, 57] or outdoors [10, 11]. This study observed a negative EB with significantly better results for those receiving high vs. low or zero CS levels. These findings suggest that supplementary feeding is required to meet the nutrient requirements of camels grazing extensively in the arid-area rangelands during the dry season; however, the effects differed depending on the level of CS.

## Conclusions

Non-supplemented camels travel longer distances with increased grazing and walking activity in arid-area rangelands with tough topography characteristics, leading to higher EE and significant weight loss. In this context, it is recommended that camels be kept in confinement and not allowed to graze, or they graze for a shorter period of time throughout the drought seasons when palatable forage is scarce. Supplementary feeding is essential to maintain camels while grazing in arid-area rangelands. When CS is utilized, the interdependent effects on forage utilization must be considered. The CS should be used under restriction or replaced partially or completely with high-quality forage during the drought season.

## Data Availability

All data and materials are owned by the authors and/or no permissions are required.

## Abbreviations

ADF: Acid detergent fiber
BW: Body weight
CP: Crude protein
CS: Concentrate supplement
DE: Digestible energy
DM: Dry matter
EB: Energy balance
ECA: Energy cost of grazing activity
EE: Energy expenditure
GE: Gross energy
GPS: Global Positioning System
HR: Heart rate
ME: Metabolizable energy
MEm: Metabolizable energy used for maintenance
NDF: Neutral detergent fiber
OM: Organic matter
RH: Relative humidity
SEM: Standard error of means
T^◦^C: Ambient temperature
THI: Temperature–humidity index
USGS: United States Geological Survey
vs.: Versus
